# Supplementary material for: EM for phylogenetic topology reconstruction on nonhomogeneous data
Source: BMC Evol Biol. 2014 Jun 17;14:132. doi: 10.1186/1471-2148-14-132 (PMC4074583; doi:10.1186/1471-2148-14-132)
Supplement: Additional file 1 — A pdf file containing figures A1, A2, and tables S1, S2 in section 1 and the explicit computation of the M-step for K81* model in section 2. [file 1471-2148-14-132-S1.pdf]

# ADDITIONAL FILE

ESTHER IBAÑEZ AND MARTA CASANELLAS

## 1. Figures and Tables

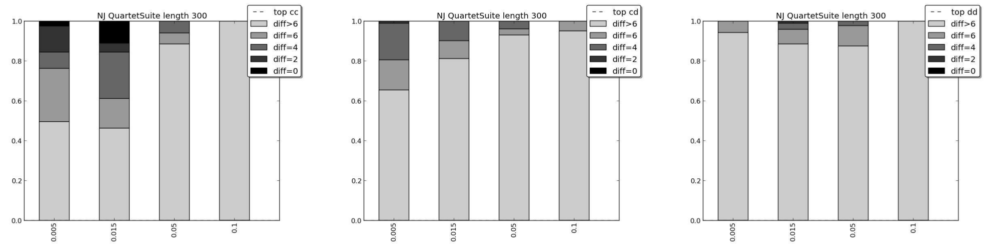


(A) QuartetSuite with `NJ` quartets as input (300 bp).

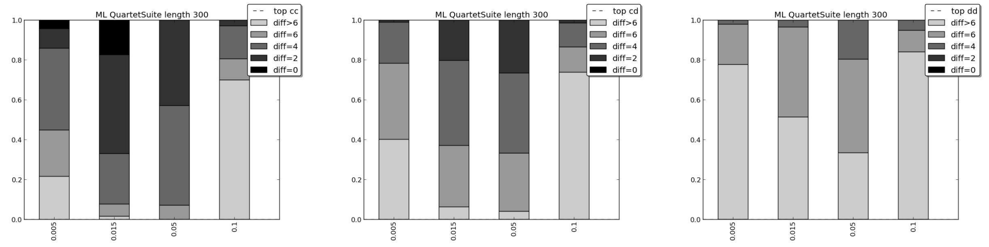


(B) QuartetSuite with `ML` quartets as input (300 bp).

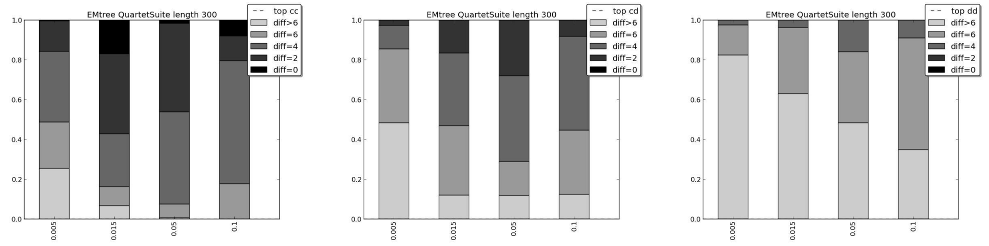


(C) QuartetSuite with `EMtree` quartets as input (300 bp).

FIGURE A1. Results of QuartetSuite for different topologies and different input methods on alignments of length 300. Each row corresponds to a different method of obtaining the quartets: `NJ` (A), `ML` (B), `EMtree` (C). Columns correspond to the three different 12-taxon topologies simulated: *cc* (left), *cd* (middle), *dd* (right).

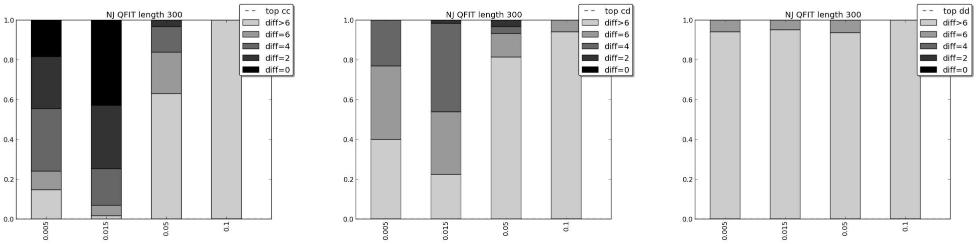


(A) QFIT with `NJ` quartets as input (300 bp).

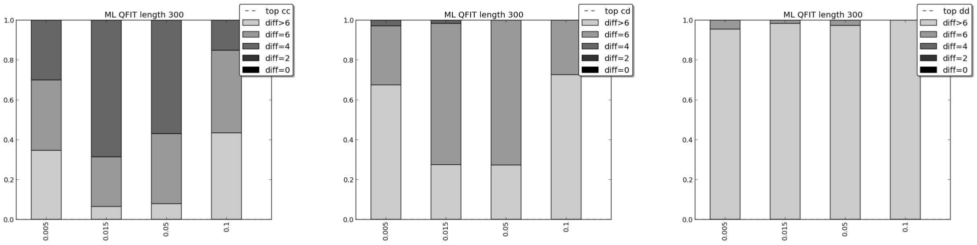


(B) QFIT with `ML` quartets as input (300 bp).

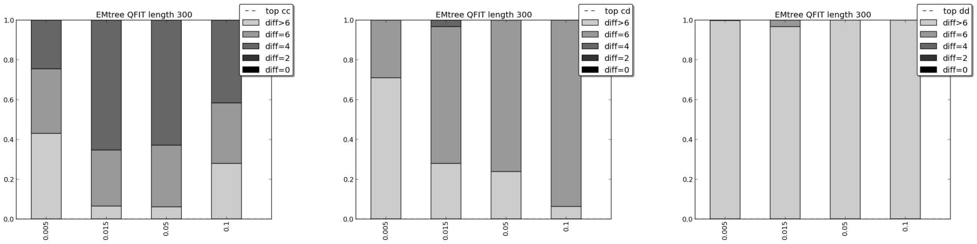


(C) QFIT with `EMtree` quartets as input (300 bp).

Figure A2. Results of QFIT for different topologies and different input methods on alignments of length 300. Each row corresponds to a different method of obtaining the quartets: `NJ` (A), `ML` (B), `EMtree` (C). Columns correspond to the three different 12-taxon topologies simulated: *cc* (left), *cd* (middle), *dd* (right).

TABLE S1. Mean of Robinson-Foulds distance with QuartetSuite. Variance is shown in brackets and $b$ refers to the branch length parameter. The length is 300 bp for the 1000 sampled alignments. The inputs for QuartetSuite have been obtained from the different methods: `NJ`, `ML`, `EMtree`. For each tree topology and each choice of parameter $b$, the best results are marked in boldface.

Mean Robinson-Foulds distance with QuartetSuite, 300 bp

| top. | Method | b | | | |
|---|---|---|---|---|---|
| | | 0.005 | 0.015 | 0.05 | 0.1 |
| *cc* | `NJ` | 7.25 (13.02) | 6.90 (18.93) | 12.43 (16.08) | 13.69 (9.33) |
| | `ML` | **5.39** (8.40) | **2.50** (2.99) | 3.28 (1.48) | 9.61 (18.33) |
| | `EMtree` | 5.54 (7.73) | 3.01 (5.07) | **3.23** (2.09) | **3.79** (2.44) |
| *cd* | `NJ` | 9.15 (15.56) | 9.94 (9.25) | 11.10 (8.58) | 13.31 (12.15) |
| | `ML` | **7.14** (7.60) | **4.46** (2.83) | **4.22** (2.83) | 10.14 (16.82) |
| | `EMtree` | 7.46 (7.58) | 4.88 (3.57) | 4.34 (4.48) | **4.98** (2.66) |
| *dd* | `NJ` | 10.82 (8.43) | 11.36 (15.65) | 11.09 (12.04) | 13.01 (8.94) |
| | `ML` | **9.14** (6.05) | **7.25** (2.51) | **6.35** (2.42) | 11.17 (14.78) |
| | `EMtree` | 9.54 (6.95) | 7.53 (2.66) | 6.76 (2.79) | **6.70** (2.35) |

TABLE S2. Mean of Robinson-Foulds distance with QFIT. Variance is shown in brackets and $b$ refers to the branch length parameter. The length is 300 bp for the 1000 sampled alignments. The inputs for QFIT have been obtained from the different methods: `NJ`, `ML`, `EMtree`. For each tree topology and each choice of parameter $b$, the best results are marked in boldface.

Mean Robinson-Foulds distance with QFIT, 300 bp

| top. | Method | b | | | |
|---|---|---|---|---|---|
| | | 0.005 | 0.015 | 0.05 | 0.1 |
| *cc* | `NJ` | **3.64** (8.04) | **1.82** (3.82) | 8.10 (8.59) | 12.73 (4.02) |
| | `ML` | 6.67 (6.20) | 4.76 (1.46) | 5.02 (1.64) | 7.14 (5.28) |
| | `EMtree` | 7.05 (6.54) | 4.82 (1.48) | **4.86** (1.46) | **5.85** (3.47) |
| *cd* | `NJ` | **6.84** (5.70) | **5.60** (3.42) | 8.99 (6.59) | 11.86 (4.61) |
| | `ML` | 8.23 (4.67) | 6.53 (0.96) | 6.55 (0.79) | 8.36 (4.39) |
| | `EMtree` | 8.54 (4.57) | 6.52 (1.15) | **6.48** (0.73) | **7.55** (3.01) |
| *dd* | `NJ` | **9.59** (3.96) | 9.26 (2.69) | 10.54 (4.82) | 11.27 (3.55) |
| | `ML` | 9.80 (3.55) | 8.37 (0.79) | 8.43 (0.89) | 9.67 (2.79) |
| | `EMtree` | 9.93 (3.29) | **8.36** (0.98) | **8.33** (0.55) | **9.05** (1.97) |

## 2. Estimation of parameters for complete data on the K81* model

Here we describe the computation of the $M-step$ of the EM algorithm for a K81* model. Let $T$ be a trivalent rooted tree topology, and denote by $\mathcal{N}$ its set of nodes and $\mathcal{E}$ its set of edges. We assume that we are given complete data, that is, a sample of observations in $\Sigma = \{A, C, G, T\}$ for all nodes of the tree. Complete data can be recorded in a vector $u_{cD}$ with $4^{|\mathcal{N}|}$ components whose coordinates are labeled by each possible observation at the set of nodes of the tree $x_1, \ldots, x_n \in \Sigma^{|\mathcal{N}|}$ and whose entries are the empirical absolute frequencies of these observations.

We define a matrix $A$ whose columns are indexed by all possible observations $x_1, \ldots, x_{|\mathcal{N}|} \in 4^{|\mathcal{N}|}$ at the nodes of $T$, and whose rows are indexed by pairs $e, (i,j)$ with $e \in \mathcal{E}$ and $(i,j) \in \Sigma^2$. At column $x_1, \ldots, x_{|\mathcal{N}|}$ and row $e, (i,j)$ where $e$ is an edge, say from node $n_1$ to node $n_2$, we set the corresponding entry of $A$ equal to 1 if $x_{n_1} = i$ and $x_{n_2} = j$, and equal to 0 otherwise.

Then it is easy to prove (using Lagrange multipliers) that the maximum likelihood estimates for the entries of the K81* substitution matrices $\{S_i\}_{i\in\mathcal{E}}$ on the tree $T$ given vector data $cD$ are:

$$(\hat{S_k})_{ii} = \frac{a_k}{a_k + b_k + c_k + d_k}, i \in \Sigma$$

$$(\hat{S_k})_{AC} = (\hat{S_k})_{CA} = (\hat{S_k})_{GT} = (\hat{S_k})_{TG} = \frac{b_k}{a_k + b_k + c_k + d_k}$$

$$(\hat{S_k})_{AG} = (\hat{S_k})_{GA} = (\hat{S_k})_{CT} = (\hat{S_k})_{TC} = \frac{c_k}{a_k + b_k + c_k + d_k}$$

$$(\hat{S_k})_{AT} = (\hat{S_k})_{TA} = (\hat{S_k})_{CG} = (\hat{S_k})_{GC} = \frac{d_k}{a_k + b_k + c_k + d_k}$$

where

$$a_k := (A\cdot u_{cD})_{k,(A,A)} + (A\cdot u_{cD})_{k,(C,C)} + (A\cdot u_{cD})_{k,(G,G)} + (A\cdot u_{cD})_{k,(T,T)}$$
$$b_k := (A\cdot u_{cD})_{k,(A,C)} + (A\cdot u_{cD})_{k,(C,A)} + (A\cdot u_{cD})_{k,(G,T)} + (A\cdot u_{cD})_{k,(T,G)}$$
$$c_k := (A\cdot u_{cD})_{k,(A,G)} + (A\cdot u_{cD})_{k,(G,A)} + (A\cdot u_{cD})_{k,(C,T)} + (A\cdot u_{cD})_{k,(T,C)}$$
$$d_k := (A\cdot u_{cD})_{k,(A,C)} + (A\cdot u_{cD})_{k,(T,A)} + (A\cdot u_{cD})_{k,(C,G)} + (A\cdot u_{cD})_{k,(G,C)}$$

for $k \in \mathcal{E}$.
